# MAGI2‐AS3 suppresses MYC signaling to inhibit cell proliferation and migration in ovarian cancer through targeting miR‐525‐5p/MXD1 axis

**DOI:** 10.1002/cam4.3126

**Published:** 2020-07-18

**Authors:** Hua Chang, Xue Zhang, Baixue Li, Xiangkai Meng

**Affiliations:** ^1^ Department of Gynecology the First Hospital of China Medical University Shenyang P.R. China; ^2^ Department of Gynecology The Third Affiliated Hospital of Guangzhou Medical University Guangzhou P.R. China

**Keywords:** MAGI2‐AS3, miR‐525‐5p, MXD1, ovarian cancer

## Abstract

Ovarian cancer (OV) is one of the most lethal gynecological malignance in females, and usually diagnosed at advanced stages. Long noncoding RNAs (lncRNAs) exhibit their crucial functions in modulatory mechanisms of cancers. Substantive studies have proven the anti‐tumor role of MAGI2‐AS3 in multiple cancers, but the physiological functions of MAGI2‐AS3 in OV need more detailed explanations. The current study corroborated that overexpression of MAGI2‐AS3 executed inhibitory activity in OV via hindering cell proliferation, cell cycle, migration as well as invasion while promoted apoptosis. Moreover MAGI2‐AS3 bound with miR‐525‐5p and negatively regulated the expression of miR‐525‐5p. Further studies testified that MXD1 was a downstream target of miR‐525‐5p and the competing relationship between MAGI2‐AS3 and MXD1 were confirmed by RNA pull down. Based on the combination between MAX and MYC, we analyzed the effects of MAGI2‐AS3 on MXD1 and MYC, unveiling the competing relationship between MXD1 and MYC for binding to MAX. Finally, we constructed rescue assays to certify that MAGI2‐AS3 suppressed the course of OV via enhancing MXD1 expression. In summary, MAGI2‐AS3 repressed the progression of OV by targeting miR‐525‐5p/MXD1 axis, offering a novel insight into understanding OV at the molecular level.

## Graphical abstract

Overexpression of MAGI2‐AS3 obstructed proliferation and motility in Ovarian cancer (OV) cells. MAGI2‐AS3 modulated the expression of miR‐525‐5p via binding to it. MXD1 was a downstream target of miR‐525‐5p in OV. MXD1 bound with MAX to repress the combination between MAX and MYC. MAGI2‐AS3 suppressed the growth of OV by elevating MXD1 expression.

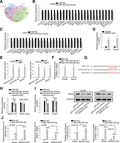

## 1 INTRODUCTION

Ovarian cancer (OV) is a malignant tumor in gynecologic pathology with a high morbidity and induces increasing deaths among the females across the global.^1^, ^2^ The primary factors contributed to late diagnosis are that there are no distinct symptoms in early stage and lack of advanced screening.^3^, ^4^ The majority of OV patients always missed the golden opportunity for surgery until the exact diagnosis. Although great improvements have been achieved in therapies for OV patients, the survival of advanced OV patients was unpromising and the prognosis within five years remained disappointing.^5^ Therefore, it is critical to get a deep understanding of latent molecular mechanism underlying OV progression.

Long noncoding RNAs (lncRNAs), over 200 nucleotides in length, are subtype of non‐coding RNAs without protein‐coding ability. Emerging literatures illustrated that lncRNAs participated in cancer initiation and progression, such as chemoresistance, metastasis and differentiation.^6^, ^7^ Importantly, numerous lncRNAs have been reported in OV. For example, lncRNA MALAT1 was highly expressed in OV cells and accelerated the invasion and proliferation in OV.^8^ LncRNA SLC27A2 affected chemoresistance of OV to cisplatin via modulating miR‐411.^9^ LINC00319 boosted OV development via targeting miR‐423‐5p/NACC1 axis.^10^ The recent studies revealed that lncRNA MAGI2‐AS3 exerted inhibitory functions in bladder cancer^11^ and breast cancer.^12^ Nevertheless, the physiological functions of MAGI2‐AS3 in OV need more specific explanations.

Competing endogenous RNA (ceRNA) mechanism was renowned for regulating gene expressions through that lncRNAs regulated the downstream targets to exert oncogenic or anti‐tumor function by sponging miRNAs.^13^, ^14^ For instance, lncRNA LUCAT1 aggravated the malignance of OV by modulating miR‐612/HOXA13 axis.^15^ LncRNA CCAT2 silence repressed oncogenic process via sponging miR‐424 in OV.^16^ LncRNA FLVCR1‐AS1 facilitated cell growth and migration in OV by mediating miR‐513/YAP1 axis.^17^ However, whether MAGI2‐AS3 mediated ceRNA mechanism in OV was not elucidated.

The purpose of our study was to identify the function and mechanism of MAGI2‐AS3 in the process of OV.

## 2 MATERIALS AND METHODS

### 2.1 Cell lines and transfection plasmids

Human OV cell lines (SKOV3, SNU119, OVCAR‐3, SUN8, Caov3) and normal ovarian epithelial cell line (IOSE) were procured from American Type Culture Collection (ATCC; Manassas, VA) and allowed to grow in the DMEM in 5% CO_2_ at 37°C. Medium was supplemented with the 10% fetal bovine serum (FBS; HyClone, Logan, UT). For transfection, the pcDNA3.1/MAGI2‐AS3 as well as pcDNA3.1/MXD1 and empty vector as negative control (NC), miR‐525‐5p mimics and miR‐NC, shRNAs specific to MXD1 (sh‐MXD1: 5'‐ CCGGGCCTCCATGTTACCATACAATCTCGAGATTGTATGGTAACATGGAGGCTTTTTG‐3') and nonspecific shRNAs as NC (termed shCtrl) (shCtrl: 5'‐ CCGGCTTACCTGATTCACCACAGATCTCGAGATCTGTGGTGAATCAGGTAAGTTTTTG‐3') as well as shRNAs specific to MAGI2‐AS3 (sh‐MAGI2‐AS3: 5'‐CCGGTTATAGGAAAGCTTTTATCTTCTCGAGAAGATAAAAGCTTTCCTATAA TTTTTG‐3') and shCtrl (shCtrl: 5'‐CCGGTTATAGGAAAGCTTTTATCTTCTCGAGAAGATAAAAGCTTTCCTATAA TTTTTG‐3'), were all synthesized at RiboBio (Guangzhou, China). Lipofectamine 2000 (Invitrogen, Carlsbad, CA) was used for conducting cell transfection in SUN8 and Caov3 cells.

### 2.2 Quantitative real‐time PCR (qRT‐PCR)

Total RNAs extracted from OV cells were achieved by Trizol reagent (Invitrogen) for converting into cDNA using Reverse Transcription Kit (Toyobo). The specific primers were synthesized by Invitrogen (18418012). SYBR Green Super Mix (Bio‐Rad) was acquired for qPCR. Results were processed by the comparative 2^−ΔΔCT^ method. GAPDH and U6 acted as the loading controls to normalize the data. All primers were listed in Table S1.

### 2.3 Cell proliferation assay

Proliferative ability of OV cells was assayed by EdU incorporation assay kit (Ribobio) after transfection. Cells were fixed and treated with 0.5% Troxin X‐100 before staining with DAPI (Beyotime) for nuclear counterstaining. Cells were observed by fluorescent microscope (Leica).

### 2.4 Flow cytometry

Cell apoptosis was quantitated by flow cytometry after double‐staining with Annexin V‐labeled with 7AAD and PE (BD Biosciences) as per the standard method. Transfected OV cells in the Binding buffer were stained in the dark for 15 min and then analyzed. To analyze cell cycle, cell samples were treated with 70% ethanol and then stained by propidium iodide (PI) and processed with flow cytometry. The proportions of cells in G0/G1, S or G2/M phase were separately determined.

### 2.5 Cell invasion and migration assay

24‐well transwell chamber (Corning) with the upper chamber coated with Matrigel (BD Bioscience) or not was used for cell invasion or migration assay. Transfected OV cells in serum‐free medium were put into the upper chamber and complete culture medium was placed into the lower chamber. After 24 hours of incubation, invading or migrating OV cells were stained with 0.1% crystal violet in methanol for counting.

### 2.6 Dual‐luciferase reporter assays

The wild‐type (WT) and mutated (Mut) miR‐525‐5p interacting sites within MAGI2‐AS3 or MXD1 were acquired to construct the luciferase reporter vectors MAGI2‐AS3‐WT/Mut and MXD1‐WT/Mut using pmirGLO Dual‐Luciferase Vector (Promega). Besides, the CDK4 promoter, CCND2 promoter, BMI1 promoter were severally PCR amplified to generate the reporter vectors pGL3‐CDK4 promoter, pGL3‐CCND2 promoter, pGL3‐BMI1 promoter using pGL3 vector (Promega). Luciferase assays were implemented via the co‐transfection of reporter vectors and indicated transfection plasmids for 48 hours and finally analyzed by dual Luciferase reporter assay system (Promega).

### 2.7 RNA pull down

Pierce Magnetic RNA‐Protein Pull‐Down Kit (Thermo Fisher Scientific) was acquired for conducting RNA pull down assay, as instructed by provider. The miR‐525‐5p‐WT and miR‐525‐5p‐Mut were biotin‐labeled via Biotin RNA Labeling Mix (Roche), then in vitro transcribed. Thereafter, the Bio‐miR‐525‐5p‐WT/Mut probes or control Bio‐NC probe were cultured with the protein extracts of OV cells and magnetic beads. Following washing, the pulled‐down compounds were detected by qRT‐PCR analysis.

### 2.8 RNA immunoprecipitation

The lysed OV cells in RNA immunoprecipitation (RIP) lysis buffer were incubated with the magnetic beads conjugated to human anti‐Ago2 antibody or normal mouse anti‐IgG antibody (Millipore). The RNAs in immunoprecipitate were extracted and purified for qRT‐PCR.

### 2.9 Western blot

Total protein samples from OV cells were used for electrophoresis on the 10% SDS‐PAGE and transfer onto the PVDF membranes. Following sealing with 5% nonfat milk, membranes were incubated with the specific primary antibodies against MXD1, MYC and control GAPDH all night, and then with appropriate secondary antibodies for 2 hours. All samples were assayed by the enhanced chemiluminescence reagent (Santa Cruz Biotechnology. Antibodies were all from Abcam.

### 2.10 Co‐immunoprecipitation (Co‐IP)

Lysates of SUN8 and Caov3 cells were acquired from the IP lysis buffer for cultivation with the antibodies against MAX and negative control IgG overnight at 4°C. After adding beads for 2 hours, the eluted protein samples were measured by western blot.

### 2.11 Statistical analysis

The experimental data were analyzed statistically by unpaired *t* tests or one‐way ANOVA using Graphpad Prism 6 software and exhibited as the mean values ± standard deviation (SD). Bio‐triplicate repeats were included in each assay. The P‐value threshold was set as 0.05 to show the statistical significance.

## 3 RESULTS

### 3.1 Overexpression of MAGI2‐AS3 obstructed cell growth and motility in OV

**FIGURE 1:**
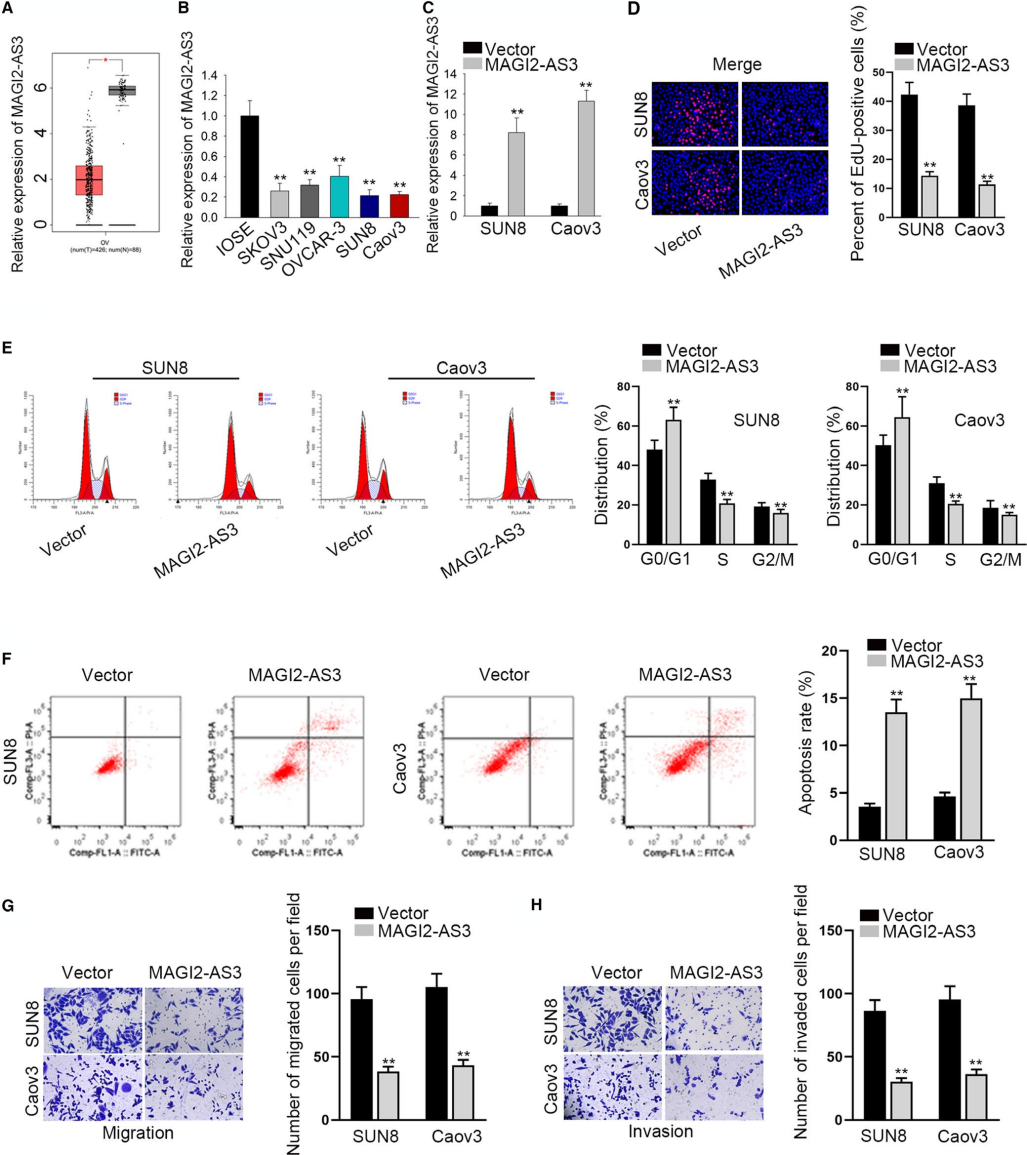
Overexpression of MAGI2‐AS3 obstructed proliferation and motility in OV cells. A, Data of GEPIA showed MAGI2‐AS3 expression in OV tissues and adjacent normal tissue. B, MAGI2‐AS3 expression in OV cell lines and IOSE cell line. C, MAGI2‐AS3 overexpression efficiency in SUN8 and Caov3 cells. D, Proliferation of MAGI2‐AS3‐overexpressed OV cells in EdU. E and F, Flow cytometry analysis of cell cycle and cell apoptosis upon MAGI2‐AS3 overexpression. (G, H) Transwell assays were applied for assessing the migration and invasion of MAGI2‐AS3‐up‐regulated cells. **P < .01

Data from GEPIA displayed that MAGI2‐AS3 showed a low expression level in OV tissues compared with adjacent normal tissue (Figure 1A). To assess the physiological function of MAGI2‐AS3 in OV, we next detected the expression of MAGI2‐AS3 in normal ovarian epithelial cell line (IOSE) and OV cell lines (SKOV3, SNU119, OVCAR‐3, SUN8, and Caov3). Consistently, MAGI2‐AS3 was lowly expressed among OV cell lines in comparison with IOSE cell line (Figure 1B). Thereafter, SUN8 and Caov3 cells presenting lower MAGI2‐AS3 expression were used for gain‐of‐function assay. SUN8 and Caov3 cells were transfected with overexpressing plasmids targeting MAGI2‐AS3, resulting in conspicuous up‐regulation of MAGI2‐AS3 expression (Figure 1C). EdU assays were adopted for evaluating cell proliferation in response to overexpression of MAGI2‐AS3. Results revealed that MAGI2‐AS3 overexpression caused a noticeable decrease of cell proliferation (Figure 1D). Besides, we also observed that overexpressed MAGI2‐AS3 arrested cell cycle transition from G0/G1 to S or G2/M phase (Figure 1E). In terms of apoptosis, data of cytometry analysis manifested that up‐regulation of MAGI2‐AS3 remarkably boosted the apoptosis of SUN8 and Caov3 cells (Figure 1F). Additionally, the migratory and invasive capacities were both dramatically declined by MAGI2‐AS3 overexpression in transwell assays (Figure 1G,H). Altogether, MAGI2‐AS3 was lowly expressed in OV cells and overexpression of MAGI2‐AS3 hindered cell growth and motility in OV.

### 3.2 MAGI2‐AS3 interacted with miR‐525‐5p in OV

**FIGURE 2:**
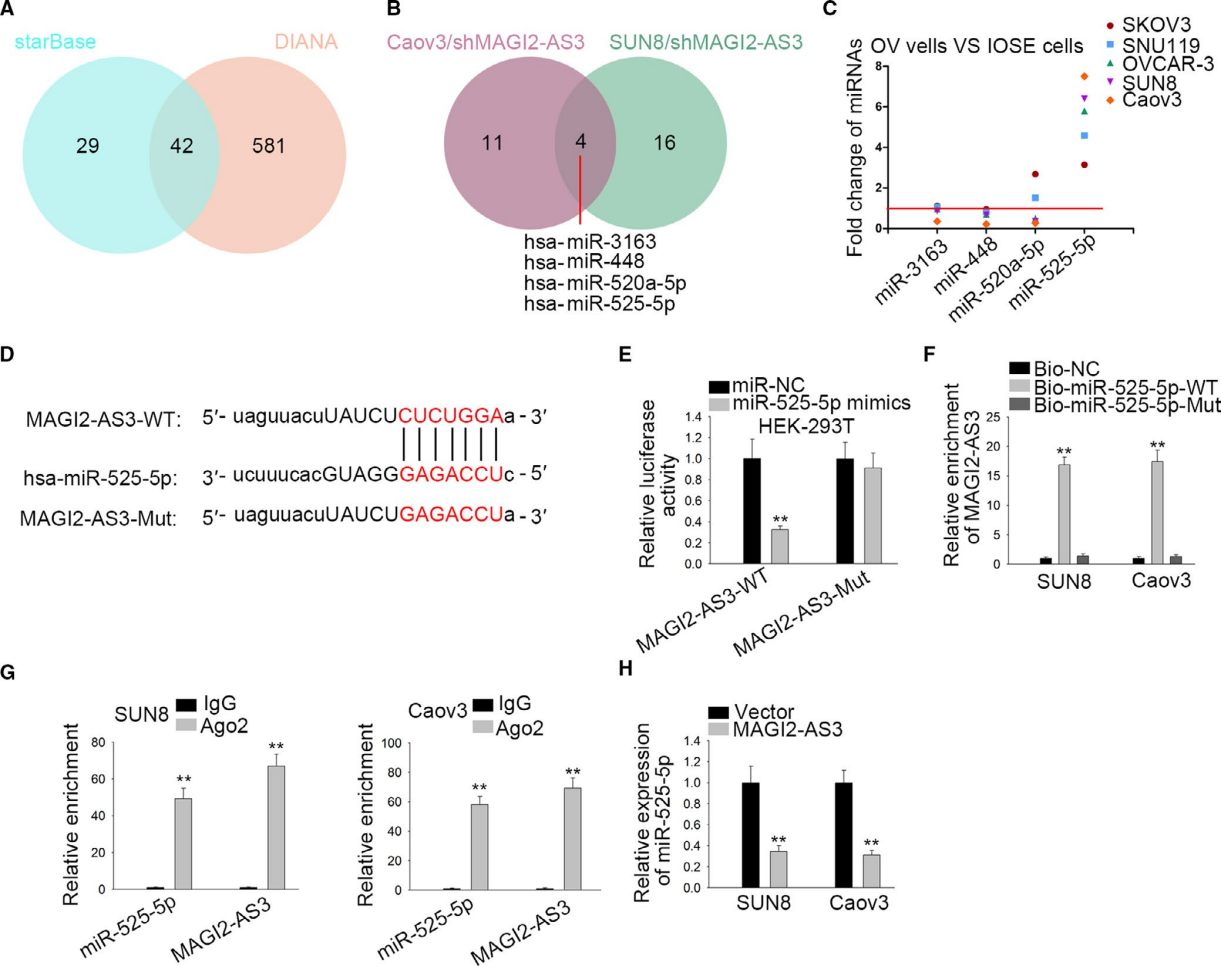
MAGI2‐AS3 sponged miR‐525‐5p in OV. A, Predicted miRNAs for MAGI2‐AS3 through combining starBase and DIANA. B, Up‐regulated miRNAs in both shMAGI2‐AS3 transfected Caov3 and SUN8 cells (C) Expressions of miR‐3163, miR‐448, miR‐520a‐5p, and miR‐525‐5p in OV cell lines. D, Bioinformatics presentation of MAGI2‐AS3 and miR‐525‐5p binding site. E, Luciferase activity in OV cells transfected with MAGI2‐AS3‐WT/Mut reporter and miR‐NC or miR‐525‐5p mimics. F, RNA pull down examined the interaction between MAGI2‐AS3 and miR‐525‐5p. G, RIP verified MAGI2‐AS3 and miR‐525‐5p co‐existed in RISCs. H, MiR‐525‐5p expression was detected after up‐regulation of MAGI2‐AS3. **P < .01

Next, we analyzed the role of MAGI2‐AS3 in ceRNA mechanism. We searched starBase (http://starbase.sysu.edu.cn/) and DIANA (http://carolina.imis.athena‐innovation.gr/diana_tools) to search miRNAs which possessed binding sites with MAGI2‐AS3. As showed in Venn diagram, there were about 42 miRNAs through combining starBase and DIANA (Figure 2A). Additionally, qRT‐PCR was performed to appraise these miRNAs expressions in SUN8 and Caov3 cells with transfecting shMAGI2‐AS3. Results displayed that expressions of miR‐3163, miR‐448, miR‐520a‐5p, and miR‐525‐5p were affected by shMAGI2‐AS3 (Figure 2B). Further, we tested expressions of above four miRNAs in OV cells and normal ovarian epithelial cell. The results disclosed that only miR‐525‐5p was highly expressed in OV cells (Figure 2C). Thus, miR‐525‐5p was predicted as a downstream gene of MAGI2‐AS3. The binding sequence between miR‐525‐5p and MAGI2‐AS3 was shown in Figure 2D. Luciferase reporter assays exhibited that the luciferase activity in OV cells transfected with MAGI2‐AS3‐WT and miR‐525‐5p mimics was decreased while no evident changes were seen in cells transfected with MAGI2‐AS3‐Mut and miR‐525‐5p mimics (Figure 2E). Results of pull down revealed that biotinylated miR‐525‐5p‐WT (Bio‐miR‐525‐5p‐WT probe) could precipitate MAGI2‐AS3 but Bio‐miR‐525‐5p‐Mut probe could not (Figure 2F). Subsequently, data of RIP showcased that MAGI2‐AS3 and miR‐525‐5p remarkably enriched in Ago2 group but not in IgG group (Figure 2G). To investigate the relationship between MAGI2‐AS3 and miR‐525‐5p, miR‐525‐5p expression was evaluated in pcDNA3.1/MAGI2‐AS3‐transfetcced SUN8 and Caov3 cells. The result displayed that miR‐525‐5p expression was diminished by MAGI2‐AS3 overexpression (Figure 2H). In short, miR‐525‐5p could bind to MAGI2‐AS3 and was negatively regulated by MAGI2‐AS3.

### 3.3 MXD1 was a downstream target of miR‐525‐5p in OV

**FIGURE 3:**
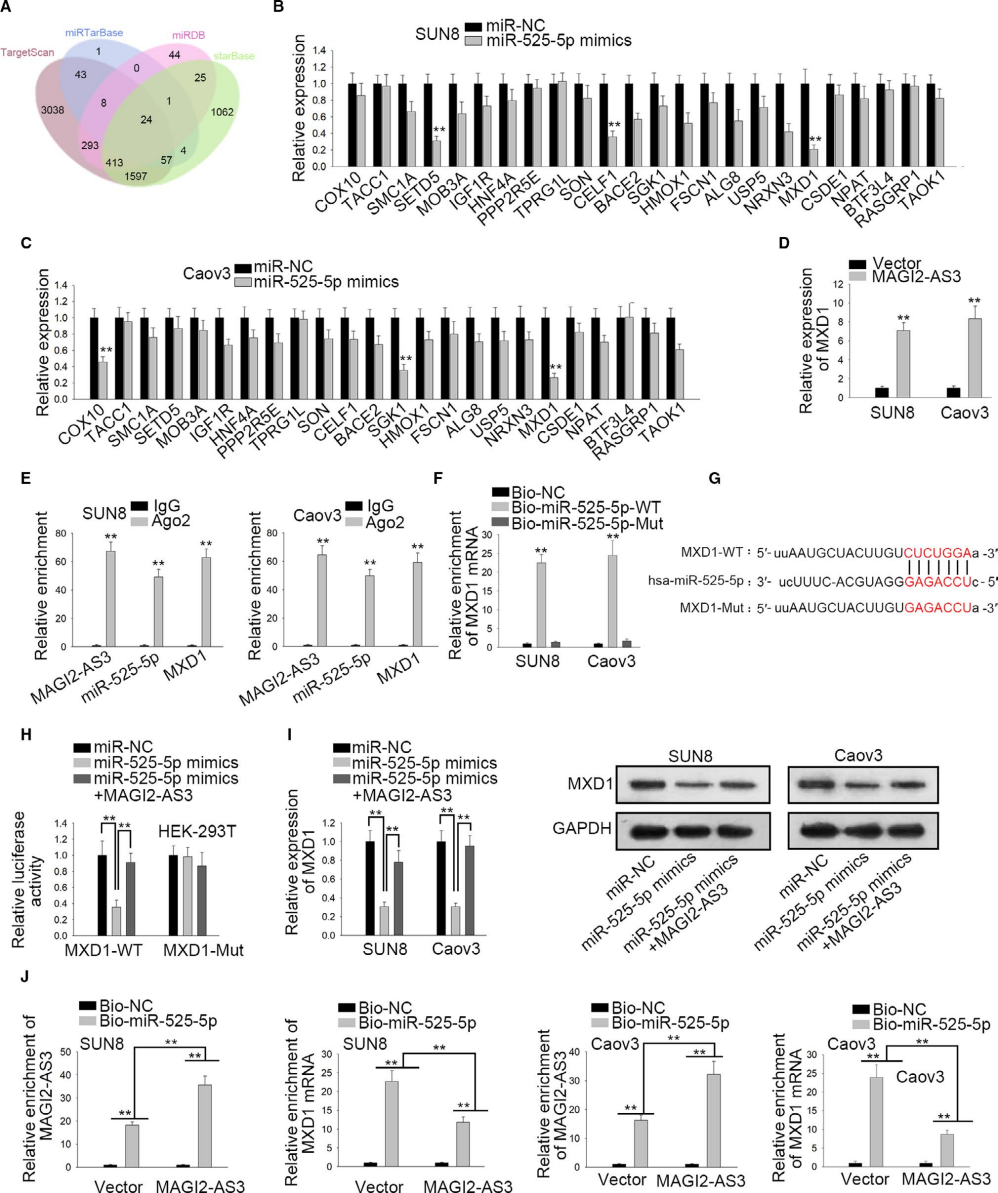
MXD1 was a downstream target of miR‐525‐5p in OV (A) Venn diagrams showed the mRNAs with binding site for miR‐525‐5p from TargetScan, miRTarBase, miRDB, and starBase. B and C, qRT‐PCR showed the expression of these mRNAs in OV cells transfected with miR‐525‐5p mimics. D, MXD1 expression was assessed in MAGI2‐AS3 up‐regulated cells. E, RIP assays demonstrated that MAGI2‐AS3, miR‐525‐5p and MXD1 co‐existed in RISCs. F, RNA pull down examined the interaction between MXD1 and miR‐525‐5p. G, Bioinformatics presentation of MXD1and miR‐525‐5p binding site. H, Luciferase activity of MXD1‐WT/Mut in response to miR‐NC, miR‐525‐5p mimics or miR‐525‐5p mimics + MAGI2‐AS3. I, qRT‐PCR and western blot examined the mRNA expression and protein level of MXD1 in cells transfected with miR‐NC, miR‐525‐5p mimics, or miR‐525‐5p mimics + MAGI2‐AS3. J, RNA pull down verified that MAGI2‐AS3 competed with MXD1 to bind with miR‐525‐5p. **P < .01

Then, we used online tools to predict the downstream targets of miR‐525‐5p. Combining TargetScan, miRTarBase, miRDB, and starBase, 24 mRNAs were predicted for miR‐525‐5p (Figure 3A). Afterwards, we performed qRT‐PCR to analyze these mRNAs expressions in miR‐525‐5p mimics‐induced SUN8 and Caov3 cells. The results hinted that only MXD1 expression was lessened in both SUN8 and Caov3 cells prominently (Figure 3B,C). Moreover we found that MXD1 mRNA and protein levels were considerably down‐regulated in OV cells through qRT‐PCR and western blot assays (Figure S1A). Besides, MXD1 was predicted to be positively associated with MAGI2‐AS3 (Figure S1B). More importantly, MXD1 expression was discovered to be elevated in MAGI2‐AS3‐overexpressed SUN8 and Caov3 cells (Figure 3D), further indicating the positive regulation of MAGI2‐AS3 on MXD1 expression in OV. Subsequently, RIP assays demonstrated that MAGI2‐AS3, miR‐525‐5p, and MXD1 co‐existed in RNA‐induced silencing complexes (RISCs; Figure 3E). RNA pull down assay delineated the notable enrichment of MXD1 in biotinylated miR‐525‐5p‐WT probe rather than biotinylated miR‐525‐5p‐Mut probe (Figure 3F). From starBase, the binding sites between MXD1 and miR‐525‐5p were predicted, and the mutation was constructed (Figure 3G). As portrayed in luciferase reporter assay, miR‐525‐5p mimics declined the activity of MXD1‐WT reporter not MXD1‐Mut reporter, but the inhibitory effect was restored by transfecting MAGI2‐AS3 (Figure 3H). Moreover qRT‐PCR and western blot revealed that MXD1 mRNA and protein expressions diminished by miR‐525‐5p up‐regulation could be lifted by overexpression of MAGI2‐AS3 (Figure 3I). Next, we carried out RNA pull down assay to study the relationship between MXD1 and MAGI2‐AS3. The consequences displayed that overexpression of MAGI2‐AS3 strengthened the interaction between miR‐525‐5p and MAGI2‐AS3 but weakened the binding of miR‐525‐5p to MXD1 (Figure 3J). In brief, MAGI2‐AS3 regulated MXD1 expression by sponging miR‐525‐5p.

### 3.4 MAGI2‐AS3 regulated MXD1 to suppress MYC

**FIGURE 4:**
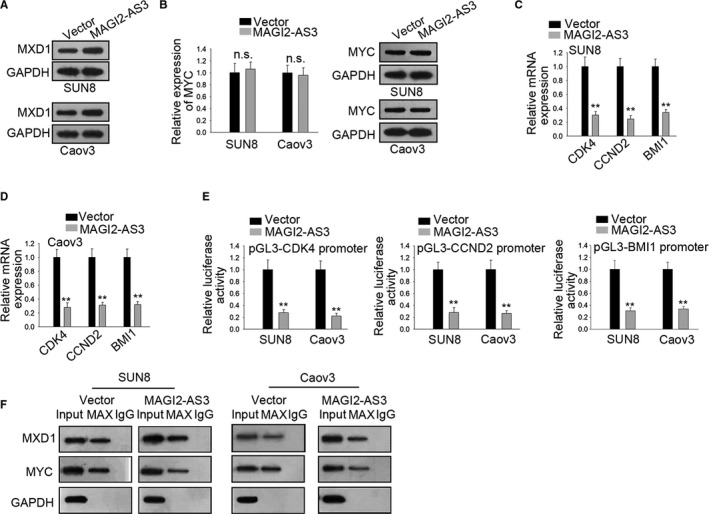
MXD1 bound with MAX to repress MYC (A) Western blot measure protein of MXD1 in MAGI2‐AS3 overexpressed cells. B, qRT‐PCR and western blot displayed the expressions of MYC mRNA and protein upon MAGI2‐AS3 up‐regulation. C and D, qRT‐PCR revealed the expressions of CDK4, CCND2 and BMI1 in MAGI2‐AS3 overexpressed cells. E, Effect of overexpressed MAGI2‐AS3 on luciferase activity of pGL3‐CDK4, pGL3‐CCND2, and pGL3‐BMI1. F, CoIP analysis showed the interaction between MAX and MXD1 or MYC. **P < .01, n.s. meant no significance

Previous studies confirmed that MXD1 could compete with MYC for the binding of MAX and thereby inhibited the transcriptional function of MYC.^18^ Thus, we supposed MAGI2‐AS3 could regulate MYC via modulating MXD1. Western blot showed that MXD1 protein was increased in cells transfected with pcDNA3.1/MAGI2‐AS3 (Figure 4A). However, there were no notable changes in MYC mRNA expression and protein level with transfection of pcDNA3.1/MAGI2‐AS3 (Figure 4B). Interestingly, we found that the expressions of MYC downstream genes (CDK4, CCND2 and BMI1) were lessened by overexpression of MAGI2‐AS3 (Figure 4C,D). Besides, the results of luciferase reporter assays displayed that the luciferase activity of these genes promoters was obviously diminished by up‐regulation of MAGI2‐AS3 (Figure 4E). Afterwards, Co‐IP analysis showed that interaction between MAX and MXD1 was increased while combination of MAX with MYC was dropped by up‐regulation of MAGI2‐AS3 (Figure 4F). To summarize, MAGI2‐AS3 could repress MYC transcriptional activity by strengthening interaction of MXD1 with MAX.

### 3.5 MAGI2‐AS3/miR‐525‐5p/MXD1 axis refrained OV cell growth and motility

**FIGURE 5:**
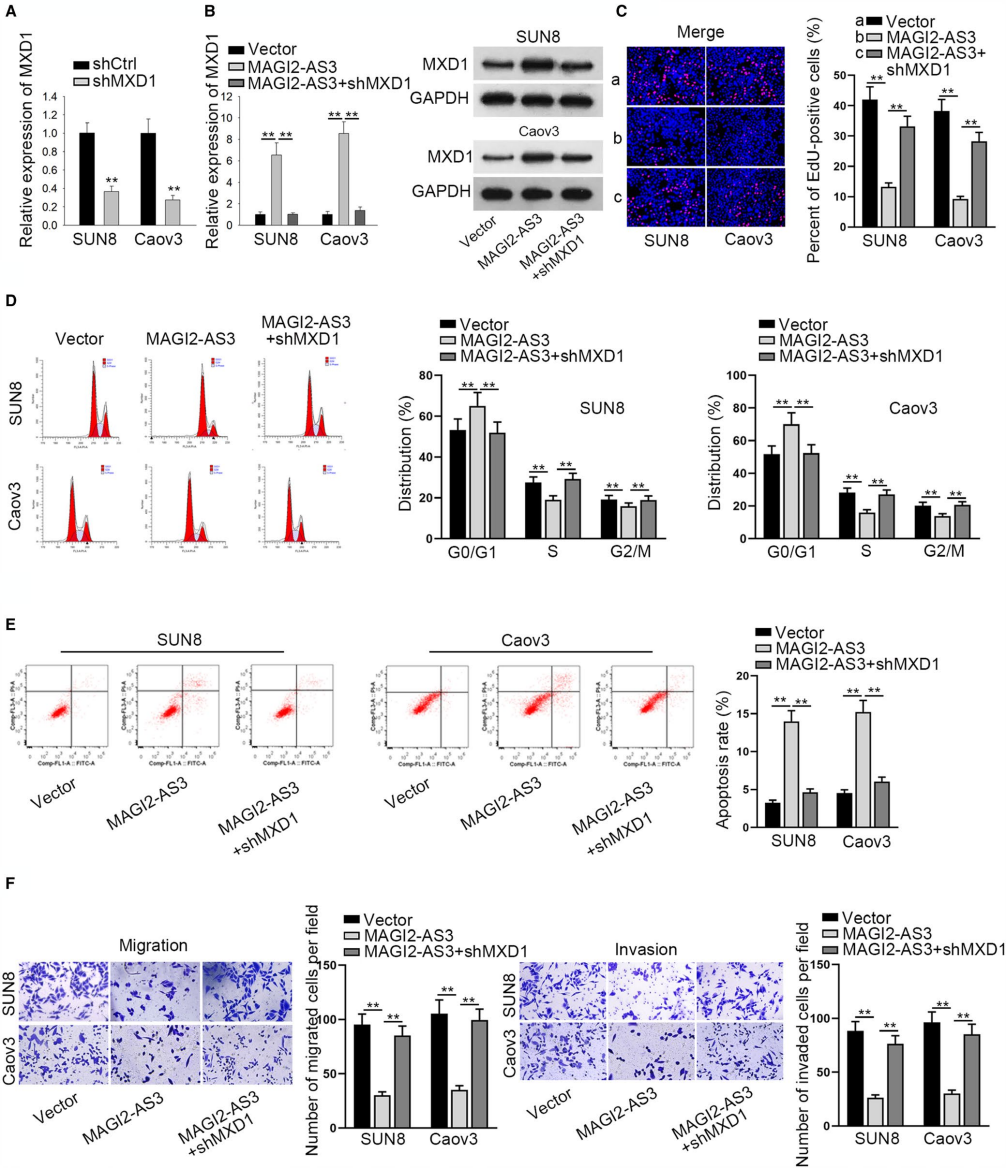
MAGI2‐AS3 suppressed the growth of OV by elevating MXD1 expression (A) qRT‐PCR revealed the expression of MXD1 in shMXD1 transfected OV cells. B, qRT‐PCR and western blot appraised the expression of MXD1 in each group. The influence of MAGI2‐AS3 on OV (C) cell proliferation, (D) cell cycle, (E) apoptosis, (F) migration and invasion was rescued by shMXD1. **P < .01

To corroborate the modulatory effect of MAGI2‐AS3 on MXD1, rescue assays were performed. To begin with, MXD1 expression was reduced by shMXD1 in SUN8 and Caov3 cells (Figure 5A). Data of qRT‐PCR and western blot revealed that MXD1 mRNA expression and protein level increased by overexpression of MAGI2‐AS3 were attenuated by knockdown of MXD1 (Figure 5B). Besides, the decreased proliferative capacity imposed by overexpression of MAGI2‐AS3 was countervailed by MXD1 knockdown (Figure 5C). Flow cytometry analysis proved that up‐regulation of MAGI2‐AS3 significantly boosted cell cycle arrest and apoptosis while silenced MXD1 offset MAGI2‐AS3 overexpression‐mediated effect (Figure 5D,E). In transwell assays, attenuated capacities of migration and invasion induced by MAGI2‐AS3 overexpression were counteracted by MXD1 down‐regulation (Figure 5F). Furthermore, the function of MAGI2‐AS3/miR‐525‐5p/MXD1 axis in OV cell growth and motility was explored in MAGI2‐AS3 overexpressed SUN8 cells (SUN8/MAGI2‐AS3). At first, we tested the transfection efficiency of pcDNA3.1/MXD1 in SUN8 cells and the overexpression efficacy was confirmed by qRT‐PCR (Figure S1C). EdU assay showed that MXD1 overexpression rescued the facilitative effect of miR‐525‐5p up‐regulation on the proliferation of SUN8/MAGI2‐AS3 cells (Figure S1D). Flow cytometry analysis manifested that promoted cell cycle and inhibited cell apoptosis caused by overexpression of miR‐525‐5p was neutralized by up‐regulation of MXD1 in SUN8/MAGI2‐AS3 cells (Figure S1E,F). According to transwell assay, we observed that overexpressed MXD1 recovered miR‐525‐5p elevation‐mediated promotion on the migration and invasion in SUN8/MAGI2‐AS3 cells (Figure S1G). Conclusively, MAGI2‐AS3/miR‐525‐5p/MXD1 axis could hinder cell growth and motility in OV.

## 4 DISCUSSION

With the development of medical level, the routine therapies for OV, including surgery, radiotherapy, and chemotherapy, have been improved. However, the overall survival rate was barely improved in the past few decades.^19^ Therefore, our study was aimed to investigate the underlying mechanism of OV.

The significant effects of lncRNAs in OV cells have been explored. For example, lncRNA HDAC1 silencing boosted the chemotherapy response in OV.^20^ LncRNA HOTAIR facilitated motility and proliferation in OV via modulating PIK3P3.^21^ LncRNA CCAT1 accelerated metastasis and predicted poor prognosis in OV.^22^ LncRNA MAGI2‐AS3 was identified to be a tumor inhibitor in breast cancer^23^ and glioma.^24^ In our study, we found that MAGI2‐AS3 was lowly expressed in OV cells and acted as a powerful factor in repressing OV development through suppressing cell proliferation, cell cycle, migration and invasion and promoting cell apoptosis in OV. Above data suggested that MAGI2‐AS3 exerted anti‐tumor effect in OV.

In terms of MAGI2‐AS3‐manipulated ceRNA mechanism in OV, we used dada from starBase and DIANA to search potential miRNAs which could bind with MAGI2‐AS3. As expected, miR‐525‐5p was found through filtration. MiR‐525‐5p was down‐regulated in cervical cancer and cancer cell proliferation and migration as well as exacerbated cell apoptosis.^25^ In the present study, miR‐525‐5p expression was in a high level in OV cells. Luciferase reporter and RNA pull down assays verified the combination between MAGI2‐AS3 and miR‐525‐5p. It was also disclosed that the expression of miR‐525‐5p was negatively regulated by MAGI2‐AS3. These results displayed that MAGI2‐AS3 sponged miR‐525‐5p in OV.

MXD1 was reported to have inhibitory functions in various cancers such as breast cancer^26^ and lung cancer.^27^ In this study, MXD1 was identified as a downstream target of miR‐525‐5p. The competition between MXD1 and MAGI2‐AS3 for the binding of miR‐525‐5p was proved by RNA pull down and luciferase reporter assay. Based on that MXD1 could inhibit MYC by competitively interacting with MAX,^18^ we assumed that MAGI2‐AS3 could directly regulate MYC expression but this hypothesis was further overturned. Then, we found that overexpressed MAGI2‐AS3 declined the expressions of CDK4, CCND2 and BMI1 which were all regulated by MYC.^28^, ^29^, ^30^ Moreover it was confirmed that MAGI2‐AS3 could contribute MXD1 to bind with MAX but inhibit MYC to bind with MAX, which suggested that MAGI2‐AS3 suppress MYC by mediating MXD1. Finally, we used rescue assays to certify that MAGI2‐AS3/miR‐525‐5p/MXD1 axis repressed OC cell growth and motility.

To sum up, our study suggested that MAGI2‐AS3 acted as a tumor inhibitor in OV through targeting miR‐525‐5p/MXD1 axis to suppress MYC signaling. This discovery will provide guidance for exploring the potential OV treatment.

## CONFLICTS OF INTEREST

Authors claim that there are no conflicts of interest in this research.

## AUTHOR CONTRIBUTION

Hua Chang: participated in the design, interpretation of the studies; Xue Zhang: conducted the experiments and analyzed the data; Baixue Li: wrote the manuscript; Xiangkai Meng critically revised the manuscript.

## ETHIC APPROVAL

Not applicable.

## Data Availability

Not applicable.
